# Fatalities

**Published:** 1905-10-15

**Authors:** 


### FATALITIES.

August 25th, a woman, 58 years old, died in a dentist's office in Keosauqua, Ia., after having five teeth extracted. Physicians who were called in stated that her death was due to Bright's disease, which was aggravated by the shock of the operation—September 2nd, a woman died in a dentist's chair at Rochester, N. Y., after having four teeth extracted. It was intended to remove fourteen teeth but her condition became alarming before the work could be completed. Her physician was present and administered the anesthetic—ether.—September 1st, a man at Upper Sandusky, O., died after the extraction of one tooth. It had been aching for some days and his face was so swollen that he could not open his mouth, so some difficulty was experienced in removing the tooth.—August 26th, a young girl at McKeesport, Pa., while delirious from pain caused by neuralgia, managed to pull out several of her teeth and died within a few hours from shock and loss of blood.—August 8th, a woman at Philadelphia, 26 years old, died from blood poisoning which followed the extraction of a tooth a few days previous.—August 4th, a woman at Altoona, Pa., 22 years old, went to a dentist to have an aching tooth extracted. He had scarcely look at the tooth when she collapsed and died almost immediately. A physician who was called assigned nervous prostration, complicated by great fear, as the cause of her death.

